# Multi‐dimensional niche differentiation of two sympatric breeding secondary cave‐nesting birds in Northeast China using DNA metabarcoding

**DOI:** 10.1002/ece3.11709

**Published:** 2024-07-07

**Authors:** Li Zhang, Zhenyun Liu, Keping Sun, Longru Jin, Jiangping Yu, Haitao Wang

**Affiliations:** ^1^ Jilin Engineering Laboratory for Avian Ecology and Conservation Genetics, School of Life Sciences Northeast Normal University Changchun China; ^2^ Jilin Provincial Key Laboratory of Animal Resource Conservation and Utilization Northeast Normal University Changchun China

**Keywords:** breeding time, diet, *Ficedula zanthopygia*, nest site, niche differentiation, *Parus minor*

## Abstract

Niche theory predicts that ecologically similar sympatric species should show differentiation in at least one of the main niche dimensions (time, space, and/or food). Here, we combined observations of breeding timing, nest site selection, and diet (the latter determined using DNA metabarcoding) to analyze the niche overlap and differentiation between two sympatric secondary cavity‐nesting birds, the Japanese Tit *Parus minor* and the Yellow‐rumped Flycatcher *Ficedula zanthopygia*. The results showed that (1) there were significant differences in the first egg laying date, length of the egg laying period, incubation date, and hatching date between tits and flycatchers, and the breeding time of flycatchers peaked later (about 30 days) than that of tits; (2) the two species had a large overlap in nest site selection, although the canopy coverage and shrub density of flycatchers were significantly higher than those of tits; and (3) the niche overlap in diet was minimal, with both species heavily relying on Lepidoptera (39.6% and 63.7% for tits and flycatchers, respectively), but with flycatchers consuming significantly higher percentages of Lepidoptera, Diptera, and Coleoptera than tits. The results indicate that these two sympatric secondary cavity‐nesting species have significant niche differentiation in breeding time and diet, but little differentiation in nest site selection.

## Graphical abstract

Two sympatric secondary cavity‐nesting bird species have significant niche differentiation in breeding time and diet.

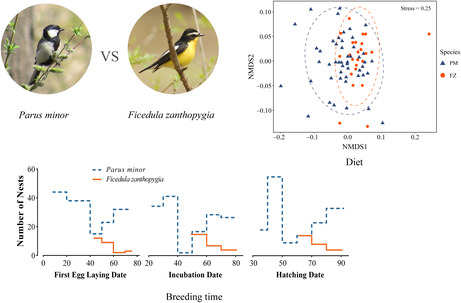

## 1 INTRODUCTION

Interspecific competition may occur when sympatric species consume or occupy a limiting resource that is critical for their survival or reproduction (Ricklefs & Schluter, 1993). Interspecific competition can lead to decreased fitness for one or multiple species involved, as they allocate resources toward competition rather than growth, reproduction, or survival. For instance, interspecific competition between great tits (*Parus major*) and collared flycatchers (*Ficedula albicollis*), which share many aspects of their ecology (e.g., nest sites and food resources), has been shown to reduce the fitness of the flycatcher by decreasing the number and quality of offspring (Gustafsson, 1987). Gause's theorem states that if two species in a community are restricted by the same resources, one will have a competitive advantage and the other will be excluded (Gause, 1934). Niche theory predicts that sympatric species with similar morphological traits and ecological requirements should show differentiation in at least one of the main ecological niche dimensions (time, space, and/or food) when in coexistence in order to diminish the negative impacts of interspecific competition and enhance fitness for these species (Cameron et al., 2007; Garcia & Arroyo, 2005; Julliard et al., 2006). Studying the degree of niche overlap and differentiation of sympatric species will help us understand the coexistence of species in ecological communities, essential knowledge for the management and conservation of species.

The breeding period in birds is part of the reproductive cycle that generally begins with egg laying. The timing of egg laying is affected by food abundance (Dias & Blondel, 1996), climate (Both & Visser, 2005), the ages of adult birds (Gonzalez‐Solis et al., 2004), photoperiod (Hahn et al., 2004), and other factors. In the breeding season, birds may require greater amounts of environmental resources due to their own requirements and those of their offspring. Specifically, in a seasonal environment, the temporal pattern of food abundance is one of the crucial factors affecting the timing of reproduction. Hence, some sympatric bird species appear to segregate breeding times to coexist stably (MacDonald et al., 2016; Ye et al., 2019). For example, the colonially nesting Little Egret (*Egretta garzetta*) and the Cattle Egret (*Bubulcus ibis*) are separated in the use of the temporal niche dimension, as the hatching time of the Little Egret peaks earlier (about 1 month) than that of the Cattle Egret (Ye et al., 2021). Considering that the timing of reproduction has a strong impact on the breeding success of birds (Nemecková et al., 2008) and that differentiation in timing plays a crucial role in species coexistence, as it is related to nest sites and food resources (Ye et al., 2019, 2021), the breeding times of sympatric species using similar ecological niches should differ (Sanz‐Aguilar et al., 2015).

The spatial overlap of habitats by bird species during the breeding season may also indicate potential interspecific competition (Friedemann et al., 2017). Nest site selection is an aspect of breeding habitat selection in bird species. Nest site selection is influenced by many factors, including nest predation, climate, competition, food resources, parasitism, and human disturbance (Chalfoun et al., 2002; Jiang et al., 2017; Maisey et al., 2016). Consequently, sympatrically breeding birds may display differentiation in nest site selection if the spatial dimension is an important aspect of niche differentiation. For example, two sympatric columbid species, the Woodpigeon (*Columba palumbus*) and the Turtle Dove (*Streptopelia turtur*), showed substantial niche differentiation in the selection of nest trees, with significant factors being the tree size and nest height (Hanane & Yassin, 2017). Competition for nest sites may be fierce for secondary cavity‐nesting bird species, as they are unable to excavate cavities by themselves but rather seek existing holes that are often limited in number. Niche differentiation in nest sites may diminish the potential for interspecific competition. However, there are few studies on niche differentiation in nest sites of sympatrically breeding secondary cavity‐nesting birds (Ye et al., 2019).

Diet similarity between species, with the addition of limited resources, has long been recognized as one of the necessary conditions for competition (Gerstell & Bednarz, 1999). Food resources during the breeding season are crucial for adult bird survival and reproductive success, including egg production, hatching success, and offspring quality (Eikenaar et al., 2003; Gerritsma et al., 2022; Peach et al., 2014; Ramsay & Houston, 1998; Ruffino et al., 2014). Adequate food resources can increase reproductive fitness. For example, food supplementation has been shown to increase the clutch size and chick survival rates of the garden‐nesting house sparrow *Passer domesticus* (Peach et al., 2014). For sympatric bird species, food consumption in large overlapping foraging habitats can intensify interspecific competition, negatively impacting their reproductive success (Minot, 1981). Birds adopt different adaptive strategies to minimize or relieve competition, including choosing different foraging habitats, foraging for different foods, or foraging during different times (Friedemann et al., 2016; Jia et al., 2019; Ye et al., 2021). For instance, the Atlantic Puffin (*Fratercula arctica*), razorbill (*Alca torda*), Common Murre (*Uria aalge*), and Black‐legged Kittiwake (*Rissa tridactyla*) breed sympatrically and overlap at foraging hotspots; however, each species is differentiated from the others in either diet (prey species, size, and number) or foraging range (Petalas et al., 2021). For sympatric and ecologically similar bird species, niche differentiation in diet can diminish competition for food resources and promote species coexistence and maintenance of biodiversity (Macarthur & Levins, 1967).

In recent years, with the advancement of high‐throughput sequencing technology, DNA metabarcoding has become increasingly utilized in wild bird dietary studies (Huang et al., 2021). This technology offers many advantages over traditional dietary analysis methods, such as nondestructive sampling, the typical use of fecal samples from study birds for analysis, and a high resolution capable of identifying prey species at the species level. For example, Bookwalter et al. (2023) used DNA metabarcoding to identify prey arthropods from the fecal samples of adult Passerine birds and compared intra‐ and inter‐species richness and diet overlap across time and space; similarly, Cabodevilla et al. (2024) used DNA metabarcoding to assess the diet of four steppe bird species.

Although some studies have been conducted on niche overlap and differentiation, relatively few studies have combined the temporal, spatial, and diet dimensions (Ye et al., 2021), resulting in a lack of comprehensive understanding concerning multi‐dimensional niche differentiation of sympatric breeding birds. In our study area, the Japanese Tit *Parus minor* and the Yellow‐rumped Flycatcher *Ficedula zanthopygia* reproduce synchronously in the same habitat, despite the tit being a resident species and the flycatcher being migratory. Both species are secondary cavity‐nesting birds and they also show a preference for artificial nest boxes for breeding. Both species mainly feed on insects in summer and have similar morphological traits and ecological requirements (Clement & de Juana, 2020; Gosler et al., 2020). Here, we combined breeding time, nest site, and diet to analyze the niche overlap and differentiation between these two species and explored the mechanism of coexistence allowing their sympatric breeding. Based on niche theory, we predicted that the Japanese Tit and the Yellow‐rumped Flycatcher would demonstrate differentiation in at least one of the niche dimensions (breeding time, nest site, and/or diet) to mitigate the negative effects of interspecific competition.

## 2 MATERIALS AND METHODS

### 2.1 Study area

The study was conducted during two breeding seasons (2021–2022) in Zuojia Nature Reserve, Jilin, northeastern China (44°1′–45°0′ N, 126°0′–126°8′ E). The reserve has an area of 60 km^2^. The altitude is 200–554.6 m, with a continental monsoon climate in the temperate zone. The average annual precipitation is 680 mm, and the average temperature is 5–6°C. The vegetation is a natural secondary broad‐leaved forest (Deng et al., 2011), including *Betula dahurica*, *Fraxinus mandschurica*, *Quercus mongolicus*, and *Tilia mandshurica*. Artificial nesting boxes (12 × 12  × 25 cm inside, 4.5 cm diameter of the hole) were hung in the study area to attract the tits and flycatchers. The nesting boxes were randomly attached to trees at a height of about 2.5–3 m above the ground, with a minimum distance of 30 m between each box. The tree species and nesting orientation were assigned at random, and the number of nesting boxes was maintained at about 450 year round (Yu et al., 2017).

### 2.2 Data collection

#### 2.2.1 Collection of breeding time data

We visited the nest boxes every 3 days after mid‐March to monitor the reproductive activities and clutch sizes of these two species (Fan et al., 2021). We recorded the first egg‐laying date, incubation date, and hatching date of the tits and flycatchers. The breeding time did not include the nestling period in this study due to the difficulty of accurately monitoring the fledging date of each nest. Breeding time data were continuously recorded for 152 and 26 nests of tits and flycatchers, respectively. In addition, to measure reproductive fitness, we recorded the brood size of both species and calculated the hatching rate (i.e., the ratio of brood size to clutch size). We collected hatching data from 57 nests of tits and 26 nests of flycatchers between mid‐May and late June when both species were breeding and experiencing interspecific competition.

#### 2.2.2 Collection of nest site data

We measured 13 nest site characteristics (Li et al., 2023) of the nest boxes occupied by tits and flycatchers. The nest height (m) (NH) above ground, the nest tree diameter (cm) at breast height (DBH), and the average diameter (cm) at breast height (ADBH) of 10 trees within a 10 m radius circle centered on the nesting tree were measured using a tape measure. The nest tree height (m) (TH), the average height (m) of 10 trees (ATH) within a 10 m radius circle centered on the nesting tree, the average height (cm) of 10 shrubs (ASH), and the shrub density (SD) within a 1 m radius circle centered on the nesting tree were measured using a laser rangefinder. We recorded the nest tree species (TS), the number of tree species (NTS), and the number of trees (NT) within a 10 m radius circle centered on the nesting tree and assessed the canopy cover (%) (CC) above the nest tree. The CC was measured by standing 1 m away from the nest tree in each of the four cardinal directions and observing it vertically to assess it (Li et al., 2023). The entrance inclination (EI) of nest boxes was measured by a digital protractor (Weidu Electronics Co., LTD; Wenzhou, China). The orientation of the nest box entrance (OE) was measured by a compass. We collected data for 300 nests of tits and 34 nests of flycatchers. The nest site data included nests with complete breeding time data, as well as those with incomplete data (i.e., missing hatching dates or nests preyed upon by predators).

#### 2.2.3 Collection of adult feces

From mid‐May to early June, adults of tits and flycatchers breeding in artificial nest boxes were captured using the baffle method after the nestlings had hatched (Fan et al., 2021). The adults were placed in a cage (18 × 12 × 8 cm) lined with sterile craft paper by an experimenter wearing sterile latex gloves. These individuals usually defecated naturally within 5 min, and the feces were collected using sterile polyester swabs and placed in lyophilizing tubes (DNase/RNase‐Free, Sterile) containing RNAlater (Servicebio, Wuhan, China). The lyophilizing tubes were gently inverted to completely immerse the feces in the RNAlater. The birds were released in place after they were ring‐banded. We obtained 49 fecal samples from adult tits across 32 nest boxes and 24 fecal samples from adult flycatchers across 19 nest boxes. The tubes containing feces were temporarily placed in a thermal bag with ice packs and then stored at −80°C until DNA extraction.

### 2.3 DNA extraction, PCR, and sequencing

Total DNA from collected fecal samples was extracted using a PowerSoil® DNA Isolation Kit (MoBio Laboratories, Carlsbad, CA, USA) according to the manufacturer's instructions. After DNA extraction, DNA integrity was tested using 1% agarose gel. We used the primers LCO‐1490 (GGTCAACAAATCATAAAGATATTGG) and ZBJ‐ArtR2c (WACTAATCAATTWCCAAATCCTCC) to amplify a 225‐bp fragment of the cytochrome c oxidase subunit I (COI) barcode region (Folmer et al., 1994; Zeale et al., 2011). Sample‐specific 8‐bp barcodes were attached to the 5′ ends of the primers. The PCR amplification was conducted in a final volume of 20 μL containing 4 μL of 5×FastPfu Buffer, 2 μL of dNTPs (2.5 mM), 0.8 μL of primer F (5 μM), 0.8 μL of primer R (5 μM), 0.2 μL of Bovine Serum Albumin (BSA), 0.4 μL of FastPfu Polymerase, and 10 ng of DNA. The conditions for PCR were as follows: an initial denaturation step at 95°C for 3 min; 95°C for 30 s, 52°C for 30 s, and 72°C for 30 s, for a total of 45 cycles, followed by a final extension at 72°C for 10 min. Each PCR batch included 1–2 PCR blanks. Three separate PCR replicates were conducted for each extract, and these were then mixed after PCR amplification. After amplification, the PCR products were visualized by electrophoresis using 1% agarose gels and purified with Agencourt AMPure XP (Beckman Coulter, Indianapolis, IN, USA). The purified products were paired‐end sequenced on an Illumina Miseq PE300 platform (Illumina Inc., SanDiego, CA, USA) by Beijing Ovison Gene Technology Co., Ltd (Beijing, China).

### 2.4 Sequencing data analysis

Raw sequences were split using QIIME v1.8.0 (Caporaso et al., 2010) based on the barcode tags. Paired‐end reads were merged and quality‐filtered by Pear v0.9.6 (Zhang et al., 2014). The sequences were processed using QIIME2 v 2020.6 (Bolyen et al., 2019). We removed duplicated sequences and chimeras using the Vsearch (Rognes et al., 2016) plugin in QIIME2, and the molecular operational taxonomic units (MOTUs) at a 97% similarity threshold were clustered (Vamos et al., 2017). We removed sequences that occurred only in a single sample and sequences with frequencies <3. The MOTUs representing <0.1% of the normalized sequences for each sample were removed to prevent the generation of potentially erroneous results (Bokulich et al., 2013). Representative sequences of each MOTU were compared with the reference sequences in the BOLD database (https://www.boldsystems.org/) and the Genbank database (https://www.ncbi.nlm.nih.gov/genbank/) to obtain taxonomic information. The taxonomic identification followed the criteria of Aizpurua et al. (2018) and Alberdi et al. (2018). Order‐level taxonomic identifications were assigned at >95% similarity values; family‐level identifications were assigned at >96.5%, and species‐level identifications were assigned at 98% similarity. All of the identified species were checked manually. When one MOTU matched multiple species that shared the highest matching score, we downgraded the taxonomic resolution to the most common level. Those MOTUs not fulfilling the criteria or not matching any reference sequence were classified as unidentified.

### 2.5 Statistical analysis

For breeding time data, the first egg‐laying date, incubation date, and hatching date were converted to Julian Days before analysis (1 April was defined as 1 each year (Fan et al., 2021)). For example, if the first egg‐laying date was 1 May, this was recorded as 31. Generalized linear mixed models (GLMMs, Poisson distribution, log link, R package lme4) were used to examine differences in breeding time variables between tits and flycatchers, with species as a fixed variable. The first egg‐laying date, incubation date, and hatching date were treated as response variables, and the year was treated as a random variable. We also calculated the breeding time overlap for each species. For example, the breeding time overlap for tits was calculated as the ratio of the co‐breeding period with flycatchers to the total breeding period for tits. Due to the abnormal distribution of hatching rates (*p* < .05), we used the Mann–Whitney U test to assess the differences between tits and flycatchers.

For nest site data, Shapiro–Wilk tests were used to examine the normality of the distributions of nest site variables. If the characteristics were normally distributed, independent‐sample *t*‐tests were used to test for differences between tits and flycatchers. Otherwise, Mann–Whitney U tests were used (Table S1).

For diet data, we computed and plotted the rarefaction and extrapolation curves of prey species richness based on Hill numbers (*q* = 0) using the R package iNEXT (Hsieh et al., 2016), developed for presence/absence data. The 95% confidence intervals (CIs) were obtained by a bootstrap method based on 1000 replications. The relative read abundance (RRA) and percent frequency of occurrence (%FOO) were used to quantify the prey composition at the order and family levels, respectively (Deagle et al., 2019). Kruskal–Wallis tests were used to test for differences in the RRA between the two species. Chi‐square tests were used to test for differences in the %FOO between tits and flycatchers; arthropods with frequency >2 were selected for the calculation. Independent‐sample *t*‐tests were used to analyze the differences in the individual diet species richness between the two bird species that were represented as the average number of arthropod species (MOTUs) consumed by each individual of each species. Nonmetric multidimensional scaling (NMDS) with Bray–Curtis distance was used to examine the degree of diet similarity between the two bird species and an analysis of similarities (ANOSIM) test with Bray–Curtis distance was used to examine the difference in diet composition between the bird species using the R package vegan (Ramette, 2007). Finally, the diet niche breadth of each species was calculated using a standardized Levins index (B_A_) (Hurlbert, 1978).

To compare the niche occupied by the tit and flycatcher across multiple dimensions, we used a multivariate kernel density estimation method to measure interspecific niche overlap (Geange et al., 2011). Initially, we calculated niche overlap between the two species for each variable. Subsequently, we obtained the composite overlap across the three major ecological dimensions (breeding time, nest site, and diet) by averaging their corresponding variables. Finally, we obtained the overall niche overlap by averaging across all variables (Wang et al., 2021). We then used null model permutation tests to determine whether an observed overlap was significantly smaller than expected by chance. For each comparison, species labels were randomized 1000 times. The overlap between simulated kernel density distributions was compared with the observed overlap using *t*‐tests. Significant differences indicated niche differentiation between the two species (Geange et al., 2011; Gotelli, 2000).

Except for the results of multivariate kernel density estimation (mean ± standard deviation), all other data were presented as mean ± standard error. All statistical analyses were conducted in R 4.1.2 (R Core Team 2022). The significance level was set at 0.05.

## 3 RESULTS

### 3.1 Breeding time overlap and differentiation

**FIGURE 1:**
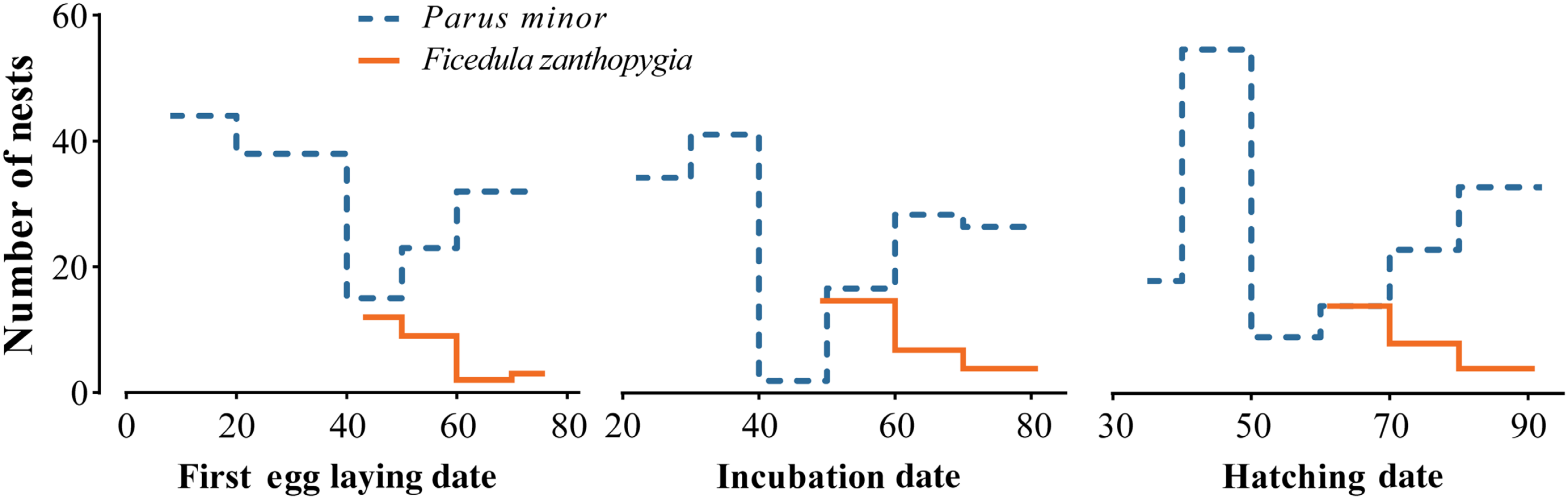
Overlap of breeding time between *Parus minor* and *Ficedula zanthopygia*.

The first egg‐laying date of tits (*n* = 152) ranged from 8 April to 12 June, while for flycatchers (*n* = 26), the date ranged from 13 May to 16 June (Figure 1). The first egg‐laying date was significantly different between bird species (GLMM, *χ* ^2^ = 29.61, *p* < .001). The incubation date of tits ranged from 22 April to 19 June, while being from 19 May to 20 June in flycatchers (Figure 1). The incubation date was significantly different between the two bird species (*χ* ^2^ = 7.33, *p* = .007). The hatching date of tits ranged from 5 May to 1 July, while for flycatchers, this ranged from 31 May to 30 June (Figure 1). There was a significant difference in hatching date between tits and flycatchers (*χ* ^2^ = 4.58, *p* = .032).

The breeding period of tits was 85 days (8 April to 1 July) and that of flycatchers was 49 days (13 May to 30 June). The percentage overlap in the breeding period was 57.64% (i.e., 49/85 days) for tits and 100% (i.e., 49/49 days) for flycatchers. The overlap of composite breeding time niches was small between tits and flycatchers. The observed overlap significantly differed from the null models, indicating niche differentiation in breeding time between the two species (Table 1). In addition, the hatching rate of tits (90.16 ± 1.42%) was significantly lower than that of flycatchers (95.60 ± 1.48%; Mann–Whitney U test; *Z* = −2. 05, *p* = .04).

**TABLE 1:** Niche overlap between *Parus minor* and *Ficedula zanthopygia* across each breeding time variable and incorporating all variables (mean ± standard deviation).

Breeding time variable	*Parus minor* vs. *Ficedula zanthopygia*
First egg‐laying date	0.448*
Incubation date	0.428*
Hatching date	0.444*
Composite	0.440 ± 0.010*

*Note*: *Parus minor* and *Ficedula zanthopygia*, which occupy significantly different niches as identified by null model tests, are indicated by “*”.

### 3.2 Nest site overlap and differentiation

In comparisons of different nest site characteristics between the two bird species, the results showed that the CC (Mann–Whitney U test; *Z* = −2.50, *p* = .012) and SD (*Z* = −3.35, *p <* .001) of flycatchers (CC: 53.53 ± 4.64%; SD: 59.06 ± 4.63%) were significantly higher than those of tits (CC: 41.44 ± 1.49%; SD: 40.91 ± 1.66%), while the other nest site characteristics were not significantly different between the two species (*p* ≥ .07 for all comparisons, Table S2). Kernel density estimation and null model tests indicated a large overlap of composite niches incorporating all 13 nest site variables between tits and flycatchers (Table 2).

**TABLE 2:** Niche overlap between *Parus minor* and *Ficedula zanthopygia* across each nest site variable and incorporating all variables (mean ± standard deviation).

Nest site variables	*Parus minor* vs. *Ficedula zanthopygia*
Nest height (cm)	0.856
Diameter at breast height (cm)	0.920
Average diameter at breast height (cm)	0.905
Nest tree height (m)	0.851
Average height of 10 trees (m)	0.847
Average height of 10 shrubs (cm)	0.873
Nest‐tree species	0.873
Number of tree species	0.806
Number of trees	0.831
Canopy cover (%)	0.801*
Shrub density (%)	0.723*
Entrance inclination (°)	0.911
Orientation entrance	0.942
Composite	0.857 ± 0.059

*Note*: *Parus minor* and *Ficedula zanthopygia*, which occupy significantly different niches as identified by null model tests, are indicated by “*”.

### 3.3 Diet composition, overlap, and differentiation

**FIGURE 2:**
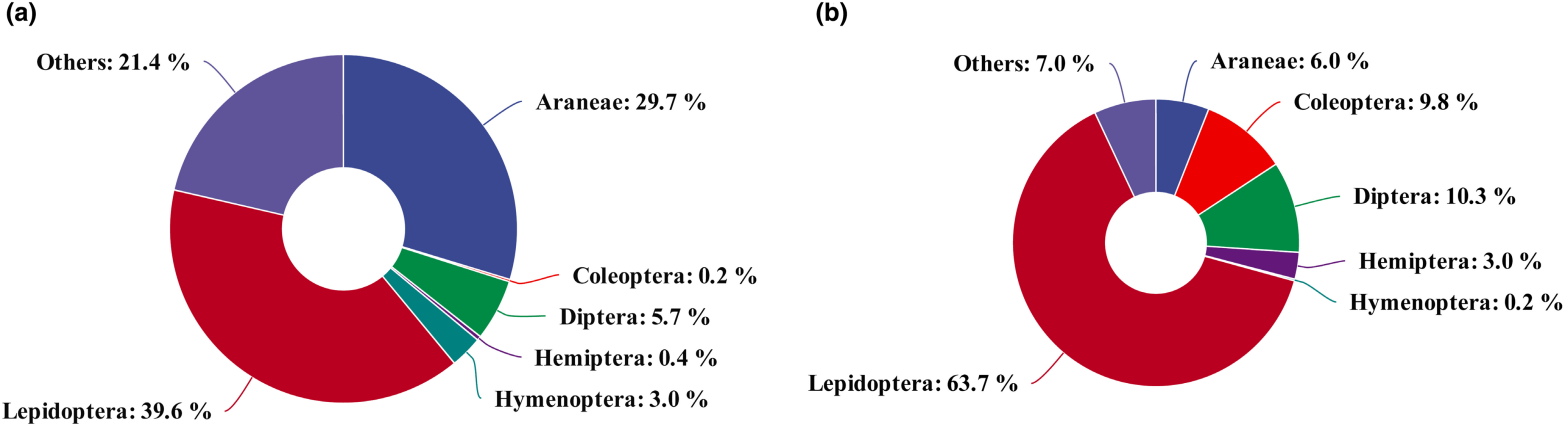
Arthropod composition and diversity in diet‐based relative read abundance (RRA) at the order level. (a) *Parus minor* and (b) *Ficedula zanthopygia*.

A total of 5,571,124 raw sequences were obtained, with an average of 76,316 sequences per sample. The rarefaction and extrapolation curves (Figure S1) based on prey species richness indicated that there was sufficient coverage of species richness for each bird species. After sequence processing and taxonomic identification, a total of 161 MOTUs were obtained through comparison with the databases, all of which were from the phylum Arthropoda. Eleven orders and 45 families were detected from the MOTUs (Table S3). The diet of tits was largely composed of Lepidoptera and Araneae, followed by Diptera and Hymenoptera (Figure 2a). The diet of flycatchers consisted primarily of Lepidoptera, followed by Diptera, Coleoptera, and Araneae (Figure 2b). The other taxa (Isopoda, Neuroptera, Noctuidae, Psocodea, and Trombidiformes) included species that appeared once or were identified at the class level (Figure 2).

**FIGURE 3:**
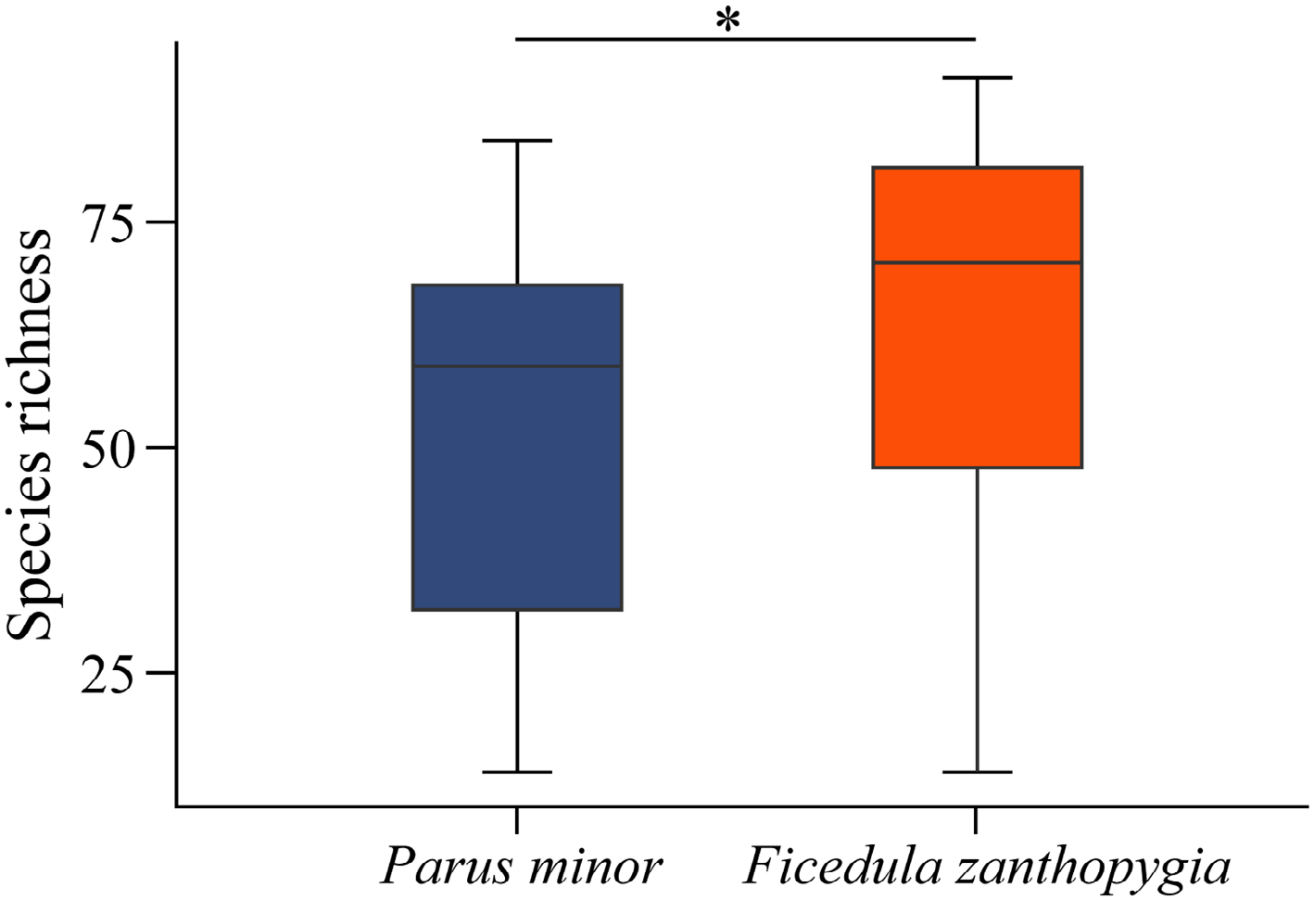
Difference of individual dietary species richness between *Parus minor* and *Ficedula zanthopygia*. Significance: *P < 0.05.

The RRA analysis of the diet composition of each bird species at the order level showed that the consumption of Lepidoptera (Kruskal–Wallis test, *χ* ^2^ = 5.23, *p* = .022), Diptera (*χ* ^2^ = 6.28, *p* = .012), and Coleoptera (*χ* ^2^ = 7.54, *p* = .006) by flycatchers was significantly higher than that by tits; the consumption of other taxa by tits was significantly higher than that by flycatchers (*χ* ^2^ = 7.36, *p* = .007). However, there were no significant differences in the consumption of Araneae, Hemiptera, or Hymenoptera between the two species (*p* ≥ .11 for all). In addition, analysis of %FOO at the family level showed that there were no significant differences in the consumption of Noctuidae, Erebidae, Geometridae, Saturniidae, or Thomisidae (Chi‐square test, *p* ≥ .33 for all); the consumption of Salticidae (*χ* ^2^ = 7.29, *p* = .007) and Tortricidae (*χ* ^2^ = 0.79, *p* = .068) by flycatchers was significantly higher than that by tits. At the MOTU level, the individual diet species richness of tits was significantly lower than that of flycatchers (Independent‐sample *t*‐test, *t* = 2.12, *p* = .037, Figure 3).

**FIGURE 4:**
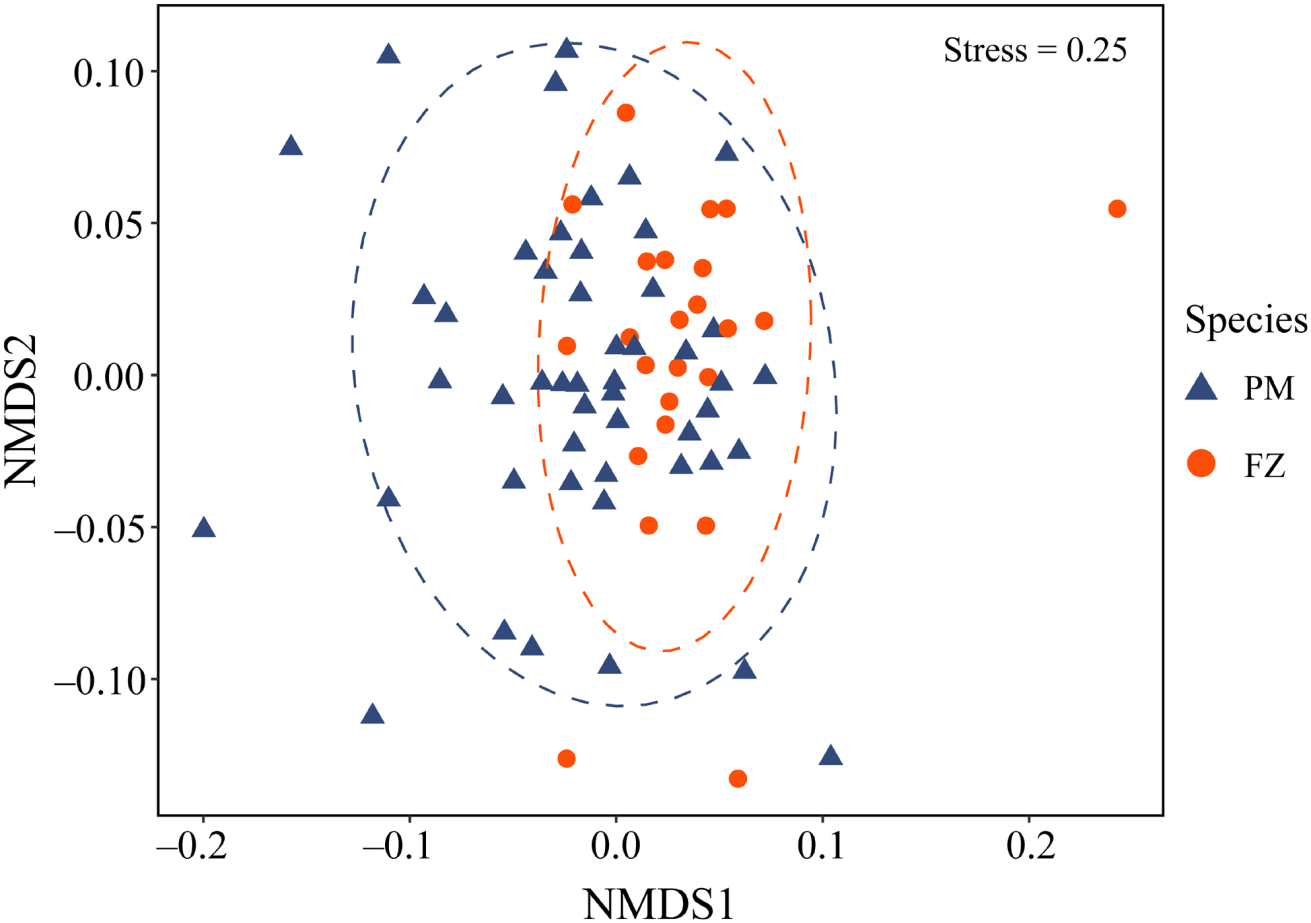
Nonmetric multidimensional scaling (NMDS) diagram showing the degree of overlap (similarity) of the diet in *Parus minor* (PM) and *Ficedula zanthopygia* (FZ). Ellipses represent 95% confidence intervals.

The NMDS analysis based on Bray–Curtis distance showed a higher degree of overlap between tits and flycatchers (Figure 4), suggesting that diet composition was similar between the two bird species. Similarly, the results of ANOSIM also showed no significant difference in the diet composition between the two species (ANOSIM test, *R* = .037, *p* = .24).

The Levins standardized measure of niche breadth showed that the diet niche breadth of flycatchers (B_A_ = 0.68) was similar to that of tits (B_A_ = 0.61). The value of diet niche overlap between the two species was slightly high, but null model tests found a significant difference between the observed overlap and the overlap expected under random assortment, indicating niche differentiation in their diet (Table 3).

**TABLE 3:** Niche overlap between *Parus minor* and *Ficedula zanthopygia* across each diet variable and incorporating all variables (mean ± standard deviation).

Diet variables	*Parus minor* vs. *Ficedula zanthopygia*
Araneae	0.895
Coleoptera	0.701*
Diptera	0.690*
Hemiptera	0.740*
Hymenoptera	0.743
Isopoda	0.943
Lepidoptera	0.742*
Neuroptera	0.743
Psocodea	0.625*
Trombidiformes	0.881
Arachnida_others	0.908
Insecta_others	0.710*
Malacostraca_others	0.341*
Composite	0.743 ± 0.156*

*Note*: *Parus minor* and *Ficedula zanthopygia*, which occupy significantly different niches as identified by null model tests, are indicated by “*.” Arachnida_others represents Arachnida prey species that were not classified at the order level; Insecta_others represents Insecta prey species that were not classified at the order level; and Malacostraca_others represents Malacostraca prey species that were not classified at the order level.

### 3.4 Overall niche overlap

Incorporating the three major dimensions of breeding time, nest site selection, and diet, we calculated the overall niche overlap between Japanese Tits and Yellow‐rumped Flycatchers as 0.763 ± 0.165. Null model tests indicated that the observed overlap was significantly smaller than expected by chance after sequential Bonferroni adjustment, indicating significant overall niche differentiation between the two species.

## 4 DISCUSSION

The differentiation in breeding time of sympatric breeding bird species plays an important role in species coexistence in the ecological community (Kronfeld‐Schor & Dayan, 2003). In our study area, the Japanese Tit is a dominant species with a relatively large population, breeding from April to July. About 30% of breeding pairs initiate a second breeding attempt during the season (Fan et al., 2021). The first egg‐laying date occurs in mid‐ to late April for the first brood and in late May for the second brood (Li et al., 2013, 2023). In contrast, the Yellow‐rumped Flycatcher, a migratory species with a smaller population, usually arrives in our study area in early May (E et al., 2017). Our results showed clear patterns of niche differentiation in breeding time between these two species. The breeding time of flycatchers peaked about 30 days later than that of tits (Figure 1). Both flycatchers and tits primarily relied on arthropods during the breeding season, and both provided their nestlings with arthropods (personal observation in the field). The differentiation in breeding time staggered the peak energy demand of nestlings to a certain extent, alleviating interspecific competition (Kushlan, 1978). Migratory birds should not arrive too early at their breeding grounds, because harsh environmental conditions, such as cold spells, may pose a mortality risk (Brown & Brown, 1998). The flycatchers arrived at our study area in early May each year when most of the tits raising the first broods were in incubation or brooding periods. When the flycatcher chicks hatched, the peak in energy demand for nestling development of tits had passed (Figure 1). Although the overlap in the breeding period was 100% for flycatchers, the number of tits raising second broods was low (Fan et al., 2021), possibly resulting in less competition. In conclusion, the results indicated that tits and flycatchers showed niche differentiation in breeding time, and this may be the result of long‐term adaptation as it could promote the coexistence of the two species.

Sympatric bird species usually experience interspecific competition for nest sites (Goldingay, 2009; Merila & Wiggins, 1995; Quintana & Yorio, 1998; Wiebe, 2016). This can promote niche differentiation to some extent (Wesołowski, 2003). In this study, we found that the Japanese Tit and Yellow‐rumped Flycatcher have a large overlap in composite nest site niches. However, the tit possessed a wider range of nest site niches than the flycatcher, except for instances where flycatchers nested in areas with greater canopy cover and shrub density. There were few natural tree holes in our study area (Deng et al., 2011); therefore, most tits and flycatchers preferred artificial nesting boxes for breeding (Zhou et al., 2009). We usually observed that tits and flycatchers competed against each other for nest boxes with similar odds of winning (personal observation, unpublished data). Our results were consistent with a previous study demonstrating that flycatchers preferred nest sites with higher tree density and canopy cover (Deng & Zhang, 2016). This preference may serve to hide the nests from detection by predators and thus reduce nest predation. Overall, the results indicated that there is intense competition for nest sites between tits and flycatchers, but the flycatchers preferred nest sites with greater nest concealment, although this may not be enough to diminish interspecific competition.

In addition to breeding time and nest site, diet niche differentiation plays a crucial role in coexistence among species (Atienzar et al., 2013; Riegert et al., 2011). Both tits and flycatchers preyed on Lepidoptera, Araneae, Diptera, Coleoptera, and Hymenoptera. However, there were significant differences in the utilization of Lepidoptera, Diptera, and Coleoptera, indicating that the two bird species had diet niche differentiation. When sympatric breeding bird species are in the breeding period with their associated high nutrient demand and are faced with possibly limited food resources, selection pressure will drive them to fulfill their energy requirement through diet differentiation. In addition, we found that tits' individual diet species richness and diet niche breadth were slightly less than those of flycatchers. Niche breadth is an index measuring the diversity of resources used by species (Sexton et al., 2017). A species that shares resources with other coexisting species has a wider niche that allows it to survive through adaptation and competition. As a migratory bird species, flycatchers usually inhabit and forage in varied environments and need to adapt to differences in prey species and abundance to fulfill their energy requirements. As a result, long‐term adaptation may enable flycatchers to take advantage of a wide range of food resources to maximize their survival and reproduction (Davis & Smith, 1998; Faria et al., 2018). Therefore, the food resources exploited by the migratory flycatchers were more diverse than those of resident tits. This result showed that there were high levels of diet overlap between tits and flycatchers, while there was some differentiation that was partly conducive to the coexistence between these two species in the breeding season.

Ecologically similar sympatric species usually show multi‐dimensional niche differentiation when coexisting. For instance, Wang et al. (2021) found significant overall niche partitioning among three sympatric pheasant species by considering microhabitat use, activity patterns, and foraging strategy. Similarly, in our study, we found clear overall niche differentiation between the Japanese Tit and Yellow‐rumped Flycatcher, which likely reduces their potential for competition and facilitates stable coexistence. Furthermore, we found that both the Japanese Tit and the Yellow‐rumped Flycatcher have high hatching rates, suggesting they have high reproductive fitness. However, hatching rates are also influenced by various factors, such as genetic effects (Briskie & Mackintosh, 2004), climate, nest type, and breeding system (Koenig, 1982; Spottiswoode & Møller, 2004). In addition, the hatching rate of the Japanese Tit was lower than that of the Yellow‐rumped Flycatcher, which may be related to their respective life history strategies (Stearns, 1976). The tit typically has two broods per breeding season, while the flycatcher has one brood in our study area. The lower hatching rate of the tit could be associated with its reduced reproductive investment or its inferior body condition during the second brood (Verhulst et al., 1997).

## 5 CONCLUSION

In this study, we found a large degree of niche overlap in the nest site between two sympatric secondary cavity‐nesting birds, the Japanese Tit and the Yellow‐rumped Flycatcher. However, notable differences were found in their breeding time and diet. These results suggest that the niche differentiation in breeding time and diet between the tit and flycatcher likely plays a crucial role in their coexistence during the breeding season. Our results are consistent with niche theory positing that sympatric species with similar morphological traits and ecological requirements can only achieve stable coexistence if they have differentiation in at least one niche dimension. This study provides important insights into the ecological mechanisms underlying the coexistence of two sympatric secondary cavity‐nest species.

## AUTHOR CONTRIBUTIONS

**Li Zhang:** Data curation (equal); formal analysis (lead); writing – original draft (lead). **Zhenyun Liu:** Data curation (equal); formal analysis (supporting); investigation (lead). **Keping Sun:** Formal analysis (supporting); methodology (equal); writing – review and editing (equal). **Longru Jin:** Conceptualization (lead); data curation (equal); funding acquisition (equal); methodology (equal); resources (equal); writing – review and editing (equal). **Jiangping Yu:** Funding acquisition (equal); resources (equal); writing – review and editing (equal). **Haitao Wang:** Funding acquisition (equal); resources (equal); writing – review and editing (equal).

## FUNDING INFORMATION

This work was supported by the Natural Science Foundation of Jilin Province, China (No. 20230101160JC to LJ) and the National Natural Science Foundation of China (No. 32371565 to JY, Nos. 31971402 and 32271560 to HW).

## CONFLICT OF INTEREST STATEMENT

The authors declare that they have no conflicts of interest.

## Supporting information


Data S1.

## Data Availability

The data are available at https://datadryad.org/stash/share/gnQCapjXix5Gi6kSAaipAbZLfU_5BWwUkjHrg8JasqA.
