# Supplementary material for: Multi‐dimensional niche differentiation of two sympatric breeding secondary cave‐nesting birds in Northeast China using DNA metabarcoding
Source: Ecol Evol. 2024 Jul 7;14(7):e11709. doi: 10.1002/ece3.11709 (PMC11227909; doi:10.1002/ece3.11709)
Supplement: Supplementary file 1 — Data S1. [file ECE3-14-e11709-s001.docx]

**Appendix**


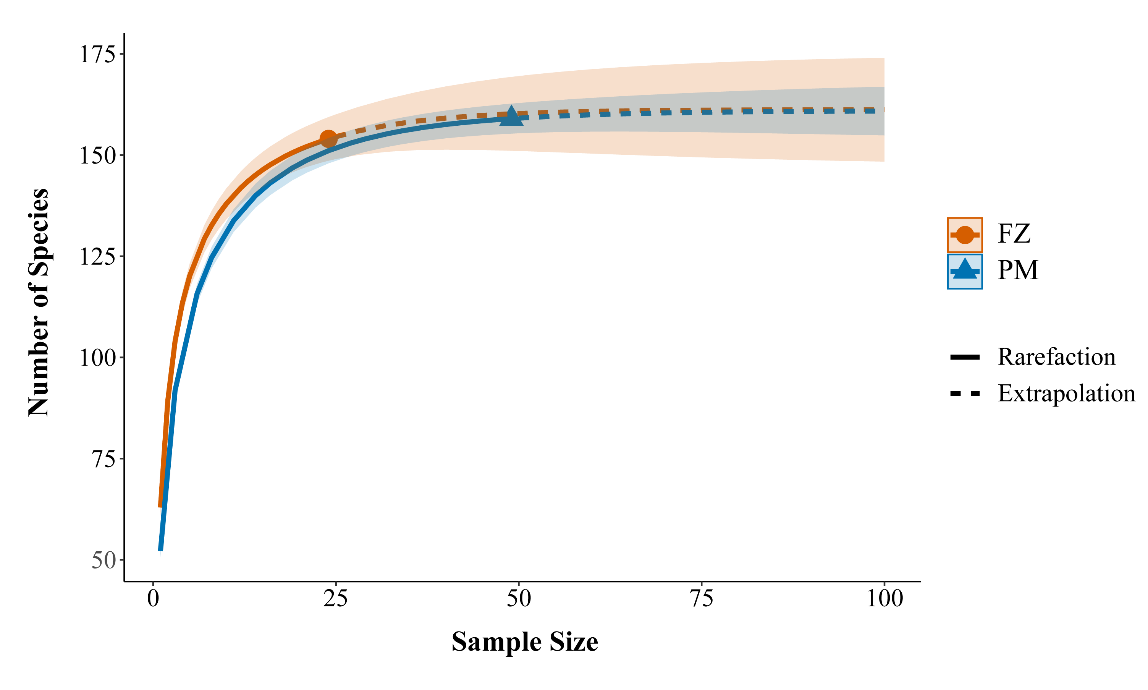


**Figure S1.** Accumulation curves of arthropod species based on the sample sizes of *Parus minor* (PM) and *Ficedula zanthopygia* (FZ).

**Table S1.** Shapiro-Wilk normality test results for nesting site characteristics.

| **Variables** | **W-value** | ***P* value** | **Distribution** |
| --- | --- | --- | --- |
| NH | 0.975 | < 0.001** | abnormal |
| DBH | 0.984 | 0.001** | abnormal |
| ADBH | 0.654 | < 0.001** | abnormal |
| TH | 0.942 | < 0.001** | abnormal |
| ATH | 0.603 | < 0.001** | abnormal |
| ASH | 0.980 | < 0.001** | abnormal |
| NTS | 0.910 | < 0.001** | abnormal |
| NT | 0.973 | < 0.001** | abnormal |
| CC | 0.963 | < 0.001** | abnormal |
| SD | 0.926 | < 0.001** | abnormal |
| EI | 0.995 | 0.389 | normal |
| OE | 0.972 | < 0.001** | abnormal |

Note: NH: Nest height; DBH: Diameter at breast height; ADBH: Average diameter at breast height; TH: Nest tree height; ATH: Average height of 10 trees; ASH: Average height of 10 shrubs; NTS: Number of tree species; NT: Number of trees; CC: Canopy cover; SD: Shrub density; EI: Entrance inclination; OE: Orientation entrance. Significance: * *P* < 0.05; ** *P* < 0.01.

**Table S2.** Differences in nest site variables between *Parus minor* and *Ficedula zanthopygia.* Data are presented as mean ± standard error.

| Variables | *Parus minor* | *Ficedula zanthopygia* | *Z* value | *P* value |
| --- | --- | --- | --- | --- |
| NH (cm) | 261.51 ± 1.70 | 262.24 ± 6.18 | –0.14 | 0.89 |
| DBH (cm) | 26.07 ± 0.24 | 25.85 ± 0.81 | –0.39 | 0.70 |
| ADBH (cm) | 25.25 ± 0.37 | 24.98 ± 1.22 | –0.04 | 0.97 |
| TH (m) | 10.19 ± 0.09 | 10.71 ± 0.29 | –1.80 | 0.07 |
| ATH (m) | 9.65 ± 0.14 | 9.65 ± 0.45 | –0.11 | 0.91 |
| ASH (cm) | 114.32 ± 3.63 | 138.26 ± 12.36 | –1.69 | 0.09 |
| TS | — | — | –0.94 | 0.35 |
| NTS | 2.83 ± 0.07 | 2.82 ± 0.17 | –0.04 | 0.97 |
| NT | 10.34 ± 0.30 | 8.94 ± 0.63 | –1.47 | 1.42 |
| CC (%) | 41.44 ± 1.49 | 53.53 ± 4.64 | –2.50 | 0.012* |
| SD (%) | 40.91 ± 1.66 | 59.06 ± 4.63 | –3.35 | <0.001** |
| EI (°) | –0.12 ± 0.36 | 0.73 ± 0.95 | 0.84 | 0.41 |
| OE | 191.06 ± 5.36 | 197.38 ± 15.06 | –0.34 | 0.73 |

Note: NH: Nest height; DBH: Diameter at breast height; ADBH: Average diameter at breast height; TH: Nest tree height; ATH: Average height of 10 trees; ASH: Average height of 10 shrubs; TS: Nest-tree species; NTS: Number of tree species; NT: Number of trees; CC: Canopy cover; SD: Shrub density; EI: Entrance inclination; OE: Orientation entrance. Significance: * *P* < 0.05; ** *P* < 0.01.

**Table S3.** Diet composition and diversity of arthropod prey at the family level of *Parus minor* (PM) and *Ficedula zanthopygia* (FZ).

| **Class** | **Order** | **Family** | **Frequency** | |
| --- | --- | --- | --- | --- |
|  |  |  | **PM** | **FZ** |
| Insecta | Lepidoptera | Noctuidae | 25 | 23 |
| Insecta | Lepidoptera | Erebidae | 10 | 9 |
| Insecta | Lepidoptera | Nymphalidae | 2 | 2 |
| Insecta | Lepidoptera | Tortricidae | 3 | 3 |
| Insecta | Lepidoptera | Nolidae | 2 | 2 |
| Insecta | Lepidoptera | Geometridae | 5 | 4 |
| Insecta | Lepidoptera | Papilionidae | 1 | 1 |
| Insecta | Lepidoptera | Saturniidae | 3 | 3 |
| Insecta | Lepidoptera | Crambidae | 1 | 0 |
| Insecta | Lepidoptera | Tineidae | 1 | 1 |
| Insecta | Lepidoptera | Depressariidae | 1 | 1 |
| Insecta | Lepidoptera | Lymantriidae | 1 | 1 |
| Insecta | Lepidoptera | Plutellidae | 1 | 1 |
| Insecta | Lepidoptera | Sphingidae | 1 | 1 |
| Insecta | Lepidoptera | Others | 14 | 13 |
| Insecta | Hymenoptera | Apidae | 2 | 2 |
| Insecta | Hymenoptera | Formicidae | 1 | 1 |
| Insecta | Hymenoptera | Ichneumonidae | 1 | 1 |
| Insecta | Diptera | Chironomidae | 2 | 2 |
| Insecta | Diptera | Drosophilidae | 1 | 1 |
| Insecta | Diptera | Culicidae | 2 | 2 |
| Insecta | Diptera | Tipulidae | 1 | 1 |
| Insecta | Diptera | Sarcophagidae | 1 | 1 |
| Insecta | Diptera | Phoridae | 1 | 1 |
| Insecta | Diptera | Tachinidae | 1 | 1 |
| Insecta | Diptera | Others | 6 | 6 |
| Insecta | Hemiptera | Aphididae | 2 | 2 |
| Insecta | Hemiptera | Urostylididae | 1 | 1 |
| Insecta | Hemiptera | Aphrophoridae | 1 | 1 |
| Insecta | Hemiptera | Others | 2 | 2 |
| Insecta | Coleoptera | Elateridae | 2 | 3 |
| Insecta | Coleoptera | Cantharidae | 2 | 2 |
| Insecta | Coleoptera | Coccinellidae | 2 | 2 |
| Insecta | Coleoptera | Nitidulidae | 1 | 1 |
| Insecta | Coleoptera | Chrysomelidae | 1 | 1 |
| Insecta | Coleoptera | Others | 1 | 1 |
| Insecta | Neuroptera |  | 1 | 1 |
| Insecta | Psocoptera | Psocidae | 1 | 1 |
| Insecta | Others |  | 11 | 11 |
| Arachnida | Araneae | Thomisidae | 9 | 9 |
| Arachnida | Araneae | Salticidae | 3 | 3 |
| Arachnida | Araneae | Agelenidae | 2 | 2 |
| Arachnida | Araneae | Philodromidae | 2 | 2 |
| Arachnida | Araneae | Araneidae | 2 | 2 |
| Arachnida | Araneae | Uloboridae | 0 | 1 |
| Arachnida | Araneae | Theridiidae | 1 | 1 |
| Arachnida | Araneae | Lycosidae | 1 | 1 |
| Arachnida | Araneae | Trachelidae | 1 | 1 |
| Arachnida | Araneae | Others | 6 | 6 |
| Arachnida | Trombidiformes | Anystidae | 1 | 1 |
| Arachnida | Others |  | 11 | 11 |
| Crustacea | Isopoda | Armadillidiidae | 1 | 1 |
| Malacostraca |  |  | 1 | 0 |
